# Genome-Wide Identification and Expression Analyses of the Fibrillin Family Genes Suggest Their Involvement in Photoprotection in Cucumber

**DOI:** 10.3390/plants7030050

**Published:** 2018-06-27

**Authors:** Inyoung Kim, Sang-Choon Lee, Eun-Ha Kim, Kihwan Song, Tae-Jin Yang, Hyun Uk Kim

**Affiliations:** 1 Department of Bioindustry and Bioresource Engineering, Plant Engineering Research Institute, Sejong University, Seoul 05006, Korea; kiy8840@naver.com (I.K.); khsong@sejong.ac.kr (K.S.); 2 Department of Plant Science, Plant Genomics and Breeding Institute and Research Institute of Agriculture and Life Sciences, College of Agriculture and Life Sciences, Seoul National University, Seoul 08826, Korea; sclee0923@phyzen.com (S.-C.L.); tjyang@snu.ac.kr (T.-J.Y.); 3 Phyzen Co., Seongnam 13558, Korea; 4 Biosafety Division, Department of Agricultural Biotechnology, National Institute of Agricultural Science, Rural Development Administration, Jeonju 54874, Korea; eunhada@korea.kr

**Keywords:** fibrillin, cucumber, genome-wide, gene expression, high light stress

## Abstract

Fibrillin (FBN) is a plastid lipid-associated protein found in photosynthetic organisms from cyanobacteria to plants. In this study, 10 *CsaFBN* genes were identified in genomic DNA sequences of cucumber (Chinese long and Gy14) through database searches using the conserved domain of FBN and the 14 *FBN* genes of Arabidopsis. Phylogenetic analysis of CsaFBN protein sequences showed that there was no counterpart of Arabidopsis and rice FBN5 in the cucumber genome. FBN5 is essential for growth in Arabidopsis and rice; its absence in cucumber may be because of incomplete genome sequences or that another FBN carries out its functions. Among the 10 *CsaFBN* genes, *CsaFBN1* and *CsaFBN9* were the most divergent in terms of nucleotide sequences. Most of the *CsaFBN* genes were expressed in the leaf, stem and fruit. *CsaFBN4* showed the highest mRNA expression levels in various tissues, followed by *CsaFBN6*, *CsaFBN1* and *CsaFBN9*. High-light stress combined with low temperature decreased photosynthetic efficiency and highly induced transcript levels of *CsaFBN1*, *CsaFBN6* and *CsaFBN11*, which decreased after 24 h treatment. Transcript levels of the other seven genes were changed only slightly. This result suggests that CsaFBN1, CsaFBN6 and CsaFBN11 may be involved in photoprotection under high-light conditions at low temperature.

## 1. Introduction

Fibrillin is a plastid lipid-associated protein found in photosynthetic organisms from cyanobacteria to plants [1,2,3,4,5,6]. It was first identified in a suborganellar structure called the fibril, which contains carotenoids, in red pepper chromoplasts [7,8]. The chromoplast fibril contains nonpolar lipids (carotenoids and tocopherol), polar lipids (galactolipids and phospholipids) and fibrillin, a 32 kDa protein [8]. Fibrillin-like proteins were subsequently reported in Japanese rose, *Nasturtium* and *Palisota barteri* [9,10]. Later, this same group of proteins were named as plastid lipid-associated proteins (PAPs), because they are located in chloroplasts and the plastids of roots, as well as chromoplasts [1]. PAP is identical to ChrB in bell pepper [7] and shows high sequence similarity with carotenoid-associated protein in cucumber chromoplasts [11] and CDSP34 [12,13], a protein that is induced under drought stress in potato chloroplasts. In addition, three fibrillin-like proteins were detected in the elaioplasts in anther, chromoplasts in petal and chloroplasts in leaf of oilseed rape (*Brassica rapa*), which were named PAPs [14]. The fibrillin isolated from pea (*Pisum sativum*) chloroplasts were named plastoglobulin (PGL) [2]. Recently, it has been suggested that these same proteins, which are called by different names, such as fibrillin, PAP and PGL, should be renamed as fibrillin (FBN) [15].

The FBN protein family has a relatively broad range of molecular weights (21–42 kDa) and isoelectric points (pI; 4–9), presumably because these proteins have a variety of biological functions [16,17]. In addition, FBNs are present in plastids, including chromoplasts, chloroplasts, elaioplasts, etioplasts and green algal chloroplast eyespots. All 14 of the FBNs in Arabidopsis are predicted to have a plastid transit peptide [16]. It was shown that different FBNs have specific functions in different plastid types and characteristic plastid structures [15,17]. However, there are few studies on the biological functions of FBN family proteins. Deruere et al. [8] demonstrated fibril reconstitution when FBN was added to a mixture of carotenoids and polar lipids. This finding indicates that FBN structurally functions in fibril assembly. In addition, FBN is either directly or indirectly related to the development of plastoglobules (PG), chromoplast pigment accumulation, hormone responses, protective photosynthetic mechanisms during light inhibition and resistance to biotic/abiotic stresses [1,4,12,13,18,19,20,21].

There are 14 FBN proteins in Arabidopsis and 7 were PG associated with thylakoids [17]. Vidi et al. [22] reported that large quantities of FBN7a (At3g58010) and FBN1a (At4g04020) were physically associated with PG in fractionation experiments. In addition, GFP fusions of these proteins were used to examine their location in the cell and a punctuate fluorescence pattern was observed in the chloroplast, which is consistent with PG-related function [22]. Through quantitative proteomics, Lundquist et al. [17] showed that FBN protein accounts for 53% of PG proteome mass. Therefore, FBN proteins generally maintain plastid lipid body structure. FBN1a, FBN1b and FBN2 were shown to function in photoprotection, mediated by ABA [20] and modulate jasmonate biosynthesis under cold stress and high light stress [21]. In Arabidopsis and apple, FBN4 is involved in biotic stress response and is related to plastoquinone content [18,19]. Recently, Arabidopsis and rice FBN5 were shown to be an essential component, along with solenesyl diphosphate synthase (SPS), in the synthesis of the solenesyl diphosphate (SPP) tail of plastoquinone-9 [23,24]. This list of the representative functions of FBN show that FBNs are involved in photosynthesis and hormone-mediated photoprotection but their specific functions still need to be studied.

Cucumber (*Cucumis sativus* L.) is a major, economically important crop belonging to the Cucurbitaceae family. Cucumber production is 192 million tons, with a land area of 9 million hectares (http://faostat.fao.org). Plants of the Cucurbitaceae family are used as model plants for studies on sex determination and vascular biology [25,26]. The first genome sequence of Cucumber was completed in 2009 [25] and in 2013, 115 wild-type cucumber genomes were sequenced, allowing comparative studies of domestication and cultivar diversity [27]. The completion of the genome made possible genome-wide analyses of multigene families of transcription factors [28,29,30,31,32] and powdery mildew MLO genes [33]. 

Cucumbers are susceptible to chilling damage because of their subtropical origin, which occurs between 0–10 °C. Chilling affects the vegetative parts as well as fruit maturity. Chilling has special effects under high-light conditions. When the temperature is reduced from 25 to 10 degrees under high-light conditions, photosynthetic efficiency drops rapidly [34,35]. It has been reported that the low-temperature sensitivity of cucumber is related to the degree of saturation of the fatty acids in the chloroplast membrane, as low temperature exposure results in decreased photosynthesis efficiency [36,37]. However, the genes involved in photoprotection under high light stress in cucumber have not been reported [38].

FBNs were recently proposed to be necessary for optimal photosynthesis [39]. To study whether the FBN family is well conserved in cucumber and their possible function during photoinhibition of photosynthesis under chilling stress, we identified the *FBN* genes in the cucumber genome databases and investigated their expression in various tissues and under high light conditions.

## 2. Results

### 2.1. FBN Family Genes in Cucumber Genomes

Through conserved domain and BLASTP searches of a cucumber genome sequence (Chinese long genome version 2) [25] from the Cucurbit Genomics Database (http://cucurbitgenomics.org/), 10 cucumber genes were predicted to encode FBN proteins (Table 1). Ten identical *FBN* genes were also identified in another cucumber genome sequence (Gy14 gynoecious inbred line) in the Phytozome 11 database (https://phytozome.jgi.doe.gov). These results from the two database searches indicated that the cucumber genome has 10 *FBN* gene family members.

Based on similarity with Arabidopsis FBNs at the amino acid level, the 10 cucumber *FBN* genes were named *CsaFBN1-11*. Among the 10 cucumber *FBN* genes, the deduced protein sequence of CsaFBN1 (Csa6M512870.1) was identical to chromoplast-specific carotenoid-associated protein CHRC (Q96398) in the corollas of cucumber [11,40]. Compared with the number of *FBN* genes in other plant species, determined using conserved domain searches, cucumber has fewer than other plant species, such as Arabidopsis, with 14 genes [15] but a similar number to others, such as rice, with 11 genes [23].

**Figure 1:**
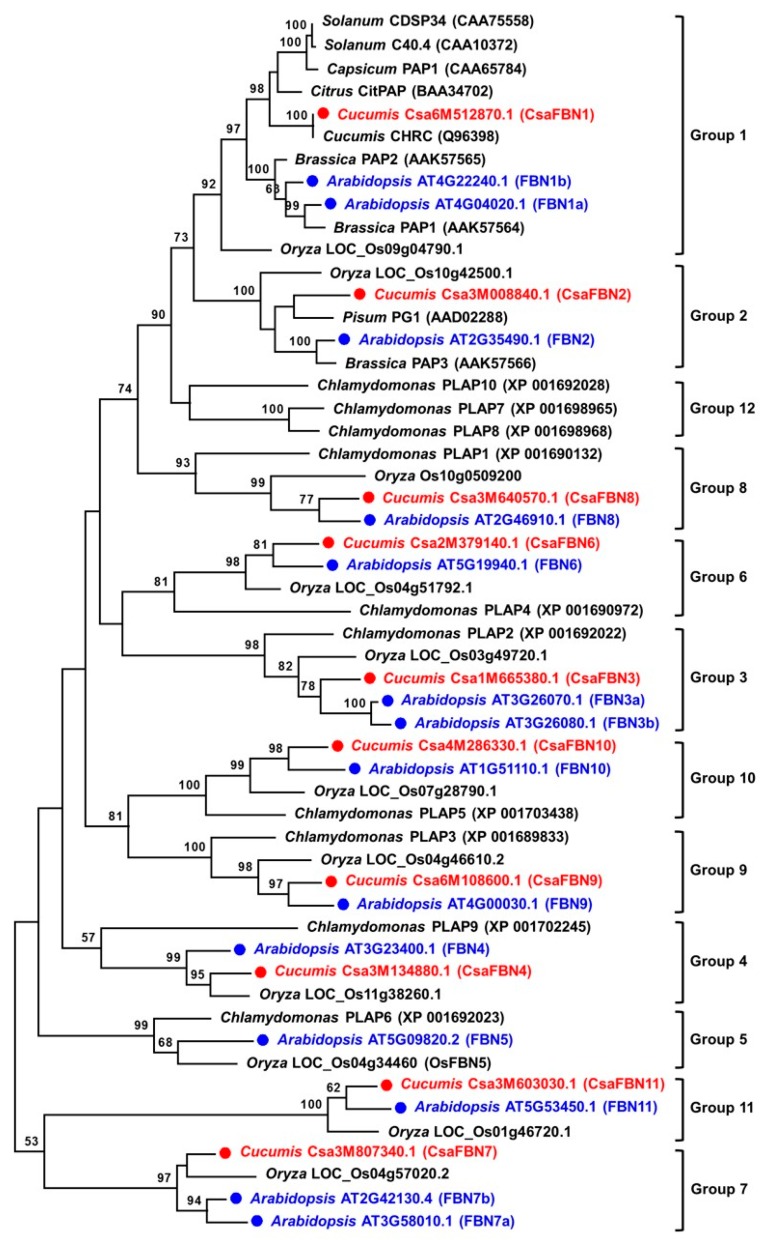
Phylogenetic relationship of FBN proteins in cucumber and other plant species. Phylogenetic tree was generated by the Maximum Likelihood (ML) method using MEGA 7.0, after alignment of deduced amino acid sequences by ClustalW. Numbers in the nodes are the bootstrap support values from 1000 replicates. Scale bar represents the number of amino acid substitution per site. *FBN* genes reported in *A. thaliana* and identified in Cucumber (this study) are shown in blue and red, respectively. Two alternative spliced forms, *Csa3M640570.2* and *Csa3M807340.2*, were not included in this tree. *FBN* genes in other plant species (*Brassica*, *Capsicum*, *Chlamydomonas*, *Oryza*, *Pisum* and *Solanum genus*) and FBN classification (Group 1 to 12) were based on Singh and McNellis [15] and Kim et al. [23].

Among the 10 *FBN* genes, the CDSs of two genes differed between Chinese long and Gy14. For one gene, *CsaFBN1*, Csa6M512870.1 (from var. Chinese long) differed from Cucsa.044900.1 (from var. Gy14) by two single nucleotide polymorphisms (SNPs) in the first exon, which resulted in a single amino acid change (Figure S1). For the second gene, *CsaFBN9*, Csa6M108600.1 (from var. Chinese long) differed from Cucsa.120630.1 (from var. Gy14) by three SNPs in the first exon, which resulted in three amino acid changes (Figure S2). The CDSs of the remaining eight *CsaFBN* genes were identical between the two varieties. Of the 10 cucumber *FBN* genes, two, *CsaFBN7* (Csa3M807340.1) and *CsaFBN8* (Csa3M640570.1), were predicted to have alternatively spliced forms, *CsaFBN7-As* (Csa3M807340.2) and *CsaFBN8-As* (Csa3M640570.2), respectively and the deduced protein sequences of the alternatively spliced forms also contained the conserved domain for plant FBN proteins (Figure 1). The CDSs of the cucumber *FBN* genes, including the alternatively spliced forms, were 636–1311 bp in size and encoded proteins of 212–437 amino acids.

### 2.2. Phylogenetic Relationships among Cucumber FBN Genes

**Figure 2:**
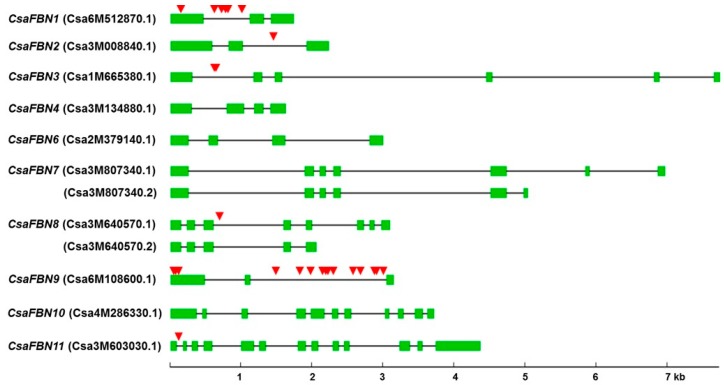
Gene structures and polymorphism of *FBN* genes in cucumber. Gene structure information of 10 cucumber *FBN* genes were retrieved from Cucurbit Genomics Database. Exons and introns are indicated by green boxes and black intervening lines, respectively. Two alternative spliced forms, *Csa3M640570.2* and *Csa3M807340.2*, were also included. Red triangles indicate polymorphic sites found between var. Chinese long and Gy14 (Table S1).

The 10 *FBN* genes identified in the cucumber genome are divided into 10 groups among the 12 groups in photosynthetic plants, including Chlamydomonas (Figure 2). *CsaFBN1* was subgrouped with *FBN1* genes expressed in fruits rather than in leaves [7,14]. The other nine genes, *CsaFBN2* to *CsaFBN11* were closely grouped with one or two Arabidopsis and rice *FBN* genes (Figure 2). These groupings were consistent with the results from the BLASTP searches against Arabidopsis *FBN* genes (Table 1). Among the 14 Arabidopsis *FBN* genes, FBN5 (AT5G09820), which is essential for the synthesis of plastoquinone-9 by interacting with SPS enzyme in Arabidopsis [24] and rice [23], had no close counterpart in cucumber (Figure 1). This was because cucumber FBN homologues of Arabidopsis genes could not be found by domain and BLAST search. In addition, no cucumber homologue of Arabidopsis FBN5 could be identified at the nucleotide or amino acid level by BLAST search against the two cucumber genome sequences or expressed sequence tags (ESTs; http://cucurbitgenomics.org/blast). Group 12 of the FBNs only existed in Chlamydomonas. This result suggests that cucumber contains 10 *FBN* genes and may have lost the *FBN5* gene, or one of the 10 FBNs performs the function of FBN5.

### 2.3. Gene Structure and Polymorphisms of FBN Genes in Cucumber

Among the 10 *FBN* genes, the genomic sequence of *CsaFBN3* (Csa1M665380.1) was the longest (7769 bp), followed by that of *CsaFBN7* (Csa3M807340.1, 6956 bp) and *CsaFBN11* (Csa3M603030.1, 4361 bp). The genomic sequence of *CsaFBN1* (Csa6M512870.1) was the shortest (1734 bp; Table 1 and Figure 2). All cucumber *FBN* genes consisted of multiple exons, ranging from 3 (*CsaFBN1* and *CsaFBN2*) to 13 (*CsaFBN11*; Figure 2). Two alternatively spliced forms, Csa3M807340.2 for *CsaFBN7* and Csa3M640570.2 for *CsaFBN8*, were shorter at the 3ʹ-termini than their corresponding normal forms.

When the genomic sequences covering the CDSs of Chinese long and Gy14 were compared, a total of 37 polymorphic sites, including 10 InDels and 27 SNPs, were found in the genomic sequences of six cucumber *FBN* genes (Figure 2 and Table S1). Most polymorphic sites were present in intron regions, except for two SNPs in the first exon region of *CsaFBN1* (Csa6M512870.1) and three SNPs in the first exon of *CsaFBN9* (Csa6M108600.1). The most variable region was the first intron of *CsaFBN9* (Csa6M108600.1), with 15 SNPs and three InDels, followed by the first intron *of CsaFBN1* (Csa6M512870.1), with six SNPs and three InDels (Table S1).

### 2.4. Expression Patterns of Cucumber FBNs in Various Tissues

**Figure 3:**
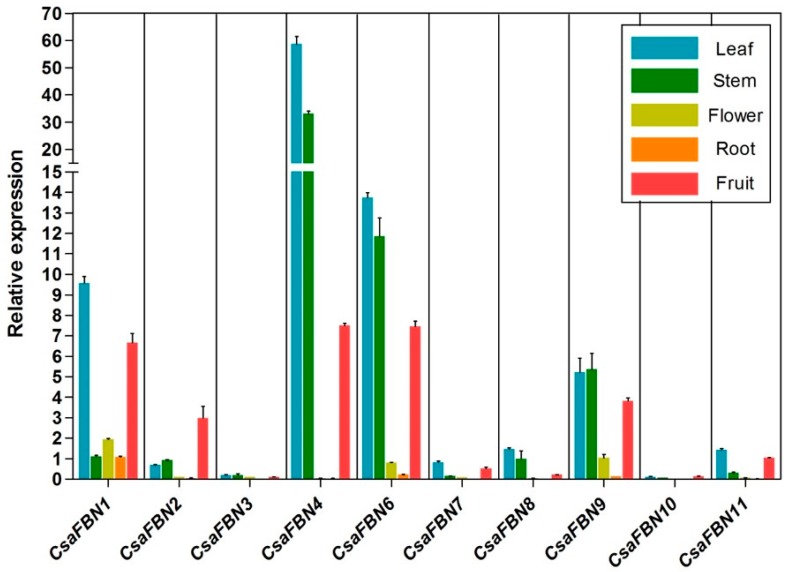
Expression profiles of cucumber *FBN* genes in various tissues. Plant tissues such as leaf, stem, flower, root and fruit were taken from cucumber plants raised for two months. Transcript expression was analyzed by quantitative real-time RT-PCR. The *EUKARYOTIC TRANSLATION INITIATION FACTOR 4A1* (eIF4a, Csa7G450710) gene from cucumber was used as an internal control. Biological triplicates were averaged. Bars indicate the standard error of the mean.

The transcriptional expression patterns of the 10 *FBN* genes in cucumber were investigated in various tissues, including, leaf, stem, flower, root and fruit, using qRT-PCR (Figure 3). To detect the expression of each *FBN* gene and to distinguish it from other members of this multigene family, gene-specific primers were designed for each gene (Table S2) and used for qRT-PCR analysis. Relative expression levels of all 10 *FBN* genes were compared with the relative expression level of *CsaFBN7* in leaf (Figure 3). All 10 *FBN* genes were expressed in all tested tissues, with varying intensities. Among the 10 genes, *CsaFBN4* showed the highest expression, followed by *CsaFBN6*, *CsaFBN1* and *CsaFBN9*, while *CsaFBN3* and *CsaFBN10* showed very low expression. *CsaFBN4* was mainly expressed in the leaf, stem and fruit but not in the flower and root. *CsaFBN6* and *CsaFBN9* were expressed in flower as well as in the leaf, stem and fruit. *CsaFBN1* transcripts were detected in all five tested tissues but higher transcript levels were detected in the leaf and fruit. *CsaFBN2* showed more predominant expression in the fruit than in other tissues. Our *FBN* expression data in cucumbers was consistent with reports from previous studies, showing that FBNs were present in leaf chloroplasts, flowers and fruit chromoplasts [2,12,14].

### 2.5. Expression of Cucumber FBNs under High Light Stress

**Figure 4:**
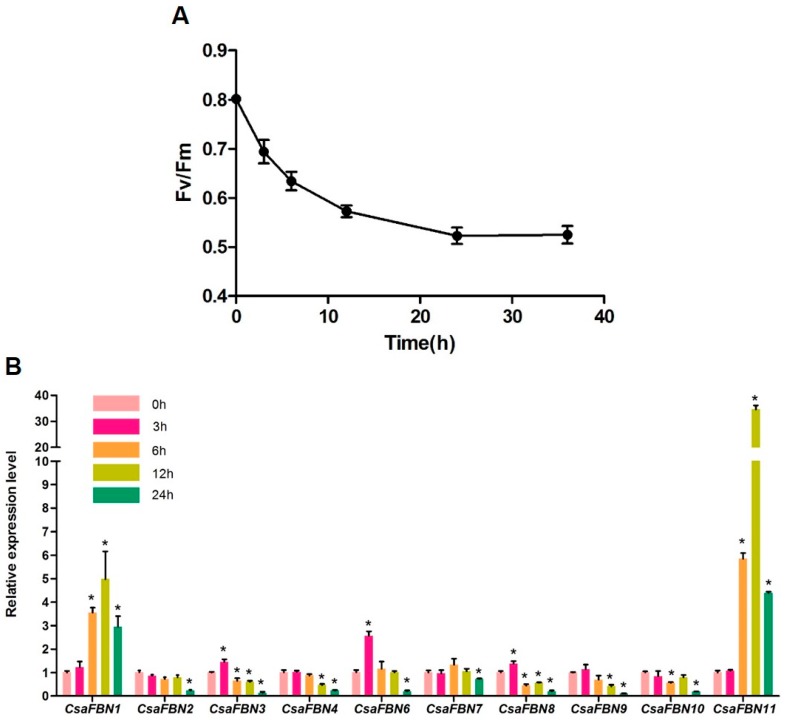
Photosynthetic capacity and *FBN*s expression under the high light with chilling stress. (**A**) Fv/Fm values of cucumber during the stressed condition (**B**) Transcript level of *CsaFBN* genes under the high-light with chilling stress. Transcript expression was analyzed by quantitative real-time RT-PCR. The *EUKARYOTIC TRANSLATION INITIATION FACTOR 4A1* (eIF4a, Csa7G450710) gene from cucumber was used as an internal control. Biological triplicates were averaged. Bars indicate the standard error of the mean. Statistically significant expression differences of gene between 0 h and high light treatment are indicated by asterisks (* *p* < 0.05, Student’s t-test).

Because the *FBN* genes in Arabidopsis, especially FBN1a, FBN1b and FBN2, are known to be involved in protection from photoinhibition under high light conditions [20,21], we investigated the expression changes in the 10 *CsaFBN* genes under high light and chilling conditions. After high light treatment, at 850 µm^−1^ and 15 °C for 3, 6, 12, 24 and 36 h, Fv/Fm was measured and leaf tissues were sampled for qRT-PCR. The Fv/Fm value represents the maximal fluorescence yield of PSII in a dark-adapted state (Fv, variable fluorescence; Fm, maximum fluorescence) and is used to assess functional damage in photosystem II [41]. The Fv/Fm of cucumber leaves gradually decreased and was steadily maintained for 24 h after treatment (Figure 4A). qRT-PCR was performed using leaf samples taken at the 0, 3, 6, 12 and 24 h time points (Figure 4B). *CsaFBN6* transcript levels increased about 2-fold after 3 h of stress treatment and then decreased. Transcripts levels of *CsaFBN1* and *CsaFBN11* did not change for up to 3 h but were highly induced at 6 h, were the highest at 12 h and then decreased to the level at 6 h by 24 h. The other seven *CsaFBN* genes showed little fluctuation in expression at 3 h and were decreased or maintained for up to 12 h of stress treatment. Expression of these genes decreased at 24 h. These results suggest that CsaFBN1, CsaFBN6 and CsaFBN11 might be involved in photoprotection in cucumber by responding to high light stress with chilling.

## 3. Discussion

FBNs are present in photosynthetic organisms and have been reported to be necessary for optimizing photosynthesis [5,20,21,24]. Cucumber is an economically important crop [25] that is known to be susceptible to low temperatures. Chilling stress of cucumber is thought to be related to fatty acid saturation in the chloroplast membrane [34]. Since the photosynthetic ability of cucumber under high light stress has not been well studied, we investigated the expression of *FBN* genes under high light stress combined with moderately cold conditions. We investigated cucumber *FBN* genes identified in a genome-wide search of two independent genome databases and only 10 genes encoding FBN proteins were identified. The genomic structure and SNP analyses of the same *FBN* genes in the Chinse long and Gy14 lines indicated that the sequences of *CsaFBN1* and *CsaFBN9* are the most diverse (Figure 2). Phylogenetic analysis showed that the 10 cucumber *FBN* genes identified in this study were divided into 10 groups, as was reported in Arabidopsis and rice [15,23], although no gene belonging to Group 5 (*FBN5*) was found in the two cucumber genomes (Table 1, Figure 1). Plant *FBN1*, *FBN2* and *FBN4* genes have been identified in many plants and have been reported to respond to abiotic stress [19,20,21]. Interestingly, *CsaFBN1*, *CsaFBN2* and *CsaFBN4* were the third, eighth and first most highly expressed, respectively, in leaves (Figure 3). A functional study of *FBN6* showed that it was the second most highly expressed in cucumber, which was not previously reported (Figure 3). Similar to cucumber *FBN* genes, most Arabidopsis *FBN* genes also showed high expression in leaves on the basis of public microarray data (Table S3).

The absence of *FBN5* in the cucumber genome is a mystery (Table 1). FBN5 is essential for plant growth, as it is a component of the SPS protein complex, which is essential for the formation of the SPP tail of plastoquinone-9 [23,24]. In Arabidopsis and rice, SPS enzymes require interaction with FBN5 for the synthesis of SPP. We identified only one SPS gene in the cucumber genome databases, SPS1 and it (Csa4G082310) shows high identity with Arabidopsis SPS1 and SPS2 as well as rice SPS2 (Figure S3), suggesting that cucumber SPS1 would also require FBN5 for efficient enzymatic activity. This speculation suggests that another FBN may fulfill the role of FBN5 in cucumber. We analyzed the pI and hydrophobicity of the FBN proteins in cucumber, Arabidopsis and rice to determine the similarity and conservation among FBN proteins in these three plant species. The results showed that most FBN orthologues were grouped by pI rather than hydrophobicity. CsaFBN6 of cucumber was found to have similar pI to Arabidopsis and rice FBN5 rather than Arabidopsis and rice FBN6 (Figure S4). When the amino acid sequences of Arabidopsis FBN5 and FBN6, rice FBN5 and CsaFBN6 were compared, CsaFBN6 showed high identity with Arabidopsis FBN6 and very interestingly, CsaFBN6 contained the conserved FV**R motif found in Arabidopsis and rice FBN5, which is absent in Arabidopsis FBN6 (Figure S5). Previously, Kim et al. [24] showed that an alternatively spliced form of Arabidopsis FBN5, which has an alteration in this region, could not interact with SPS. Thus, we were tempted to suggest that CsaFBN6 may interact with SPS and function similar to FBN5 in Arabidopsis and rice [23,24]. However, whether CsaFBN6 plays a role in SPS activity in cucumber requires confirmation in vitro and in vivo.

Comparison of the expression levels of the 10 *FBNs* of cucumber under high light and chilling stress showed that most of the *CsaFBN* genes tended to decrease or fluctuate very little with the decrease in photosynthetic ability (Figure 4). However, the expression levels of *CsaFBN1, CsaFBN6* and *CsaFBN11* increased and was maintained at high levels during stress. These results suggest that *CsaFBN1*, *CsaFBN6* and *CsaFBN11* are the main *FBN* genes that respond to light stress combined with chilling. Similarly, Arabidopsis *FBN1* (AT4G04020) and *FBN6* (AT5G19940) were also regulated by cold and light stress (Tables S4 and S5). On the other hand, unlike *CsaFBN4*, Arabidopsis *FBN4* (AT3G23400) was highly up-regulated by light stress (Table S5).

Future investigations of their roles during stress would be exciting. Characterization of the *FBN* genes in cucumber and their expression under high light stress with chilling provides the basic information that could be used to develop cucumber resistant to high light with chilling stress.

## 4. Materials and Methods 

### 4.1. Plant Materials and Growth Conditions

To analyze *FBN* gene expression, a Korean semi-white type cucumber inbred line (SJ170) provided by Dr. Kihwan Song (Sejong University, South Korea), was used. Cucumber was grown in soil under a 16 h/8 h light/dark photoperiod, with 100 µmol m^–2^ s^–1^ fluorescent light, at 23 °C, as normal conditions. For high light stress combined with low temperature stress, six-week-old plants were transferred to growth chambers, with 850 µmol m^–2^ s^–1^ fluorescent light, at 15 °C.

### 4.2. Identification of the FBN Genes in Cucumber Genomes

Two approaches were used to find *FBN* family genes in cucumber genomes. First, the conserved domain in plant FBN was used in a search. For this search, two sets of deduced protein sequences (25,600 and 21,503) from two cucumber genomes were respectively downloaded from the Cucurbit Genomics Database (Chinese long genome version 2, http://cucurbitgenomics.org/organism/2) [25,42] and Phytozome 11 database (Gy14 gynoecious inbred line, https://phytozome.jgi.doe.gov/pz/portal.html#!info?alias=Org_Csativus) and then analyzed by InterProScan (ver. 5.16-55.0, https://www.ebi.ac.uk/interpro/, EMBL-EBI, Cambridgeshire, UK) using the Pfam option to search for conserved domains. Protein sequences with plastid lipid-associated protein/FBN conserved domain (IPR006843, http://www.ebi.ac.uk/interpro/entry/IPR006843) were selected. In the second approach, we searched for sequences homologous to Arabidopsis FBN proteins. For this approach, the deduced protein sequences of 14 Arabidopsis *FBN* genes [15] were used as queries in BLASTP searches against the deduced protein sequence sets in the cucumber genome database (Chinese long genome version 2, http://cucurbitgenomics.org/blast; Gy14 gynoecious inbred line, https://phytozome.jgi.doe.gov/pz/portal.html#!search?show=BLAST&method=Org_Csativus). We then selected cucumber protein sequences with scores >200 from the BLASTP results and used in a search for the conserved domain of plant FBN using the InterPro web-based search tool (https://www.ebi.ac.uk/interpro/) and CD search tool (https://www.ncbi.nlm.nih.gov/Structure/cdd/wrpsb.cgi). Finally, cucumber genes encoding the conserved plant FBN domain were selected for further study. The chromosomal location and genome structure of selected genes were analyzed based on gene annotation information provided in the Cucurbit Genomics Database.

### 4.3. Phylogenetic Analysis of FBN Genes

The deduced protein sequences of the cucumber *FBN* genes were aligned with those of Arabidopsis and other plants [15,23] by ClustalW in MEGA 7.0 (https://www.megasoftware.net/) [43]. Then, a phylogenetic tree was generated by the Maximum Likelihood (ML) method with 1000 bootstrap replicates using MEGA 7.0.

### 4.4. Sequence Polymorphism Analysis of the FBN Genes in Two Cucumber Varieties

The genomic sequences containing the coding sequences (CDSs) of the cucumber FBNs were retrieved from the two cucumber genome databases for the varieties Chinese long and Gy14 gynoecious inbred line. The sequences of each FBN gene were aligned using NCBI Global Align (https://blast.ncbi.nlm.nih.gov/Blast.cgi) and polymorphic sites were investigated.

### 4.5. Quantitative Real-Time RT-PCR Analysis

The leaves of a cucumber plant were sampled at 0, 3, 6, 12, 24 and 36 h after high light treatment under low-temperature conditions and tissues, including leaf, stem, flower, root and fruit, were sampled from plants raised for two months. Total RNAs were extracted using the Plant RNA kit (Qiagen, Germany) and treated with RNase-free DNase I to remove genomic DNA. Then, cDNA was synthesized from the total RNA using the cDNA Synthesis kit (Takara, Japan) according to the manufacturer’s recommendations. Real-time PCR was performed with SYBR Green premix and the primers used for real-time qRT-PCR are listed in Table S2. The expression values for each *CsaFBN* gene was normalized relative to the CsaeIF4a (Csa7G450710) gene. Relative expression levels from the qRT-PCR reactions, which were performed with biological triplicates using total RNA samples extracted from three independent replicate samples, were evaluated by the comparative 2^−ΔΔCT^ method [44].

### 4.6. Measurement of Photosynthetic Parameters

The maximal fluorescence yield of PSII (Fv/Fm) was measured with a Fluorpen FP 100 (PSI, Drasov, Czech). Plant leaves were dark adapted for 15 min before measuring using leaf clips and then light was provided at 3000 µmol m^–2^ s^–1^. Fv/Fm was calculated as follows: (Fm − Fo)/Fm, where Fm is the maximal chlorophyll fluorescence intensity and Fo is the minimal chlorophyll fluorescence intensity. Measurements were obtained in four repetitions from six individual plants.

## 5. Conclusions

In this study, 10 *CsaFBN genes* were identified in two cucumbers, Chinese long and Gy14. Cucumber contains orthologues of all Arabidopsis and rice *FBN* genes, except for *FBN5*. The 10 cucumber *FBN* genes are expressed at different levels in various tissues but their expression levels were high in the leaf and fruit tissues. High light with chilling stress decreased the photosynthetic capacity of cucumber and under these conditions, transcript levels of *CsaFBN1*, *CsaFBN6* and *CsaFBN11* were highly induced. This study provides information for functional characterization of the FBNs in cucumber.

## Figures and Tables

**Table 1:** *FBN* genes identified in cucumber genome.

Gene Name	Gene ID	Location on Chromosome ^(5)^	CDS Length (bp)	Protein (aa)	Fibrillin Domain	Best Arabidopsis Homologue	% ID	E-Value
					(IPR006843) (Cdd:pfam04755)			
*CsaFBN1*	Csa6M512870.1 ^(1)^ (Cucsa.044900.1 ^(2)^)	Chr6 (26485594..26487860)	969	323	98–312	AT4G04020.1 (FBN1a, FIB1a, AtPGL35)	60	5.1 × 10^−96^
*CsaFBN2*	Csa3M008840.1 (Cucsa.322030.1)	Chr3 (992602..995095)	1092	364	139–353	AT2G35490.1 (FBN2, FIB2, AtPGL40)	51	5.7 × 10^−77^
*CsaFBN3*	Csa1M665380.1 (Cucsa.027640.1)	Chr1 (26931385..26939153)	732	244	74–233	AT3G26070.1 (FBN3a, FIB3a)	69	2.0 × 10^−71^
*CsaFBN4*	Csa3M134880.1 (Cucsa.255550.1)	Chr3 (9072862..9075038)	870	290	91–286	AT3G23400.1 (FBN4, FIB4)	62	1.0 × 10^−83^
*CsaFBN6*	Csa2M379140.1 (Cucsa.161880.1)	Chr2 (19193933..19197291)	747	249	79–244	AT5G19940.1 (FBN6, FIB6)	58	1.3 × 10^−72^
*CsaFBN7*	Csa3M807340.1 (Cucsa.242700.1)	Chr3 (30901949..30909200)	912	304	97–280	AT2G42130.4 (FBN7b, FIB7b)	72	2.2 × 10^−97^
	Csa3M807340.2 (As ^(3)^)	Chr3 (30901949..30907204)(As)	819 (As)	273 (As)	97–243	AT2G42130.1 (FBN7b, FIB7b)	72	8.1 × 10^−82^
*CsaFBN8*	Csa3M640570.1 (Cucsa.149720.1)	Chr3 (25041843..25045492)	837	279	58–270	AT2G46910.1 (FBN8, FIB8)	63	1.6 × 10^−76^
	Csa3M640570.2 (As)	Chr3 (25042753..25045492)(As)	636 (As)	212 (As)	58–194	AT2G46910.1 (FBN8, FIB8)	66	2.6 × 10^−53^
*CsaFBN9*	Csa6M108600.1 (Cucsa.120630.1)	Chr6 (7303911..7307440)	642	214	39–204	AT4G00030.1 (FBN9, FIB9)	77	2.2 × 10^−72^
*CsaFBN10*	Csa4M286330.1 (Cucsa.153490.1)	Chr4 (11046106..11050268)	1311	437	99–275	AT1G51110.1 (FBN10, FIB10)	67	7.2 × 10^−129^
*CsaFBN11*	Csa3M603030.1 (Cucsa.040680.1 ^(4)^)	Chr3 (23336459..23340819)	1773	590	359–443	AT5G53450.1 (FBN11, AtFib11)	66	9.3 × 10^−216^

^(1)^ Gene id in cucumber genome database in Cucurbit Genomics database (http://cucurbitgenomics.org/organism/2); ^(2)^ Gene id in cucumber genome database in Phytozome 11 (https://phytozome.jgi.doe.gov/pz/portal.html#!info?alias=Org_Csativus); ^(3)^ As, alternative spliced form predicted in Cucumber genome annotation v2 (http://cucurbitgenomics.org/organism/2); ^(4)^ The gene in cucumber genome database in Phytozome 11 encodes 664 amino acids, which is 74 aa larger than those encoded by Csa3M603030.1; ^(5)^ Location on chromosome, position of mRNA predicted in cucumber genome v2 (http://cucurbitgenomics.org/organism/2).

## Supplementary Materials

Supplementary materials can be found at http://www.mdpi.com/2223-7747/7/3/50/s1. Figure S1: Comparison of deduced amino acid sequences of CsaFBN1 (Csa6M512870.1) in var. Chinese long with that (Cucsa.044900.1) in var. Gy14, Figure S2: Comparison of deduced amino acid sequences of CsaFBN9 (Csa6M108600.1) in var. Chinese long with that (Cucsa.120630.1) in var. Gy14, Figure S3: Protein sequence alignment among Arabidopsis SPS1, SPS2, cucumber SPS1 and rice SPS2, Figure S4: Physicochemical properties of FBN proteins from Arabidopsis, rice and cucumber. PI and hydrophobicity (GRAVY index) were measured for each FBN after removal of the chloroplast transit peptide using the ProtParam tool (ExpPASy), Figure S5: Protein sequence alignment among Arabidopsis FBN6, rice FBN5, Arabidopsis FBN5 and cucumber FBN6, Table S1: Polymorphic sites found in genomic sequences of cucumber fibrillin genes, Table S2: Oligonucleotides Used in this Study, Table S3: Expression profiles of Arabidopsis FBN genes in various tissues and developmental stages, Table S4: Expression profiles of Arabidopsis *FBN* genes under cold stress, Table S5: Expression profiles of Arabidopsis *FBN* genes under light stress.

## Abbreviations

FBN	Fibrillin
PAPs	Plastid lipid-associated proteins
PGL	Plastoglobulin
