# Supplementary material for: Genome-Wide Identification and Expression Analyses of the Fibrillin Family Genes Suggest Their Involvement in Photoprotection in Cucumber
Source: Plants (Basel). 2018 Jun 27;7(3):50. doi: 10.3390/plants7030050 (PMC6161074; doi:10.3390/plants7030050)
Supplement: Supplementary file 1 [file plants-07-00050-s001.zip › Figures S1-S5_Plants.pdf]

```
Csa6M512870.1      MAFVSQFNQLPCKTLALNPPQPQLTSKPSVFPIASIGATARAAAGKSLISVRPAFKVRAV
Cucsa.044900.1     MAFVSQFNQLPCKTLALNPPQPQLTSKPSVFPIASIGATARAAAGKSLVSVRPAFKVRAV
                   ************************************************:***********

Csa6M512870.1      LNDDEWGEDKDEKYGDDSSVAVAEKEEEKPLEPSEIYKLKKALVDSFYGTDRGLRVSRDT
Cucsa.044900.1     LNDDEWGEDKDEKYGDDSSVAVAEKEEEKPLEPSEIYKLKKALVDSFYGTDRGLRVSRDT
                   ************************************************************

Csa6M512870.1      RAEIVELITQLESKNPTPAPTEALTLLNGKWILAYTTFAGLFPLLSRNLPLVKVEEISQT
Cucsa.044900.1     RAEIVELITQLESKNPTPAPTEALTLLNGKWILAYTTFAGLFPLLSRNLPLVKVEEISQT
                   ************************************************************

Csa6M512870.1      IDSENLTVQNSVQFSGPLATTSITTNAKFEVRSPLRVHIKFEEGVIGTPQLTDSIVIPDN
Cucsa.044900.1     IDSENLTVQNSVQFSGPLATTSITTNAKFEVRSPLRVHIKFEEGVIGTPQLTDSIVIPDN
                   ************************************************************

Csa6M512870.1      VDFLGQKIDFTPFNGIISSLQDTASNVAKTISSQPPIKFSISNTRVESWLLTTYLDEDLR
Cucsa.044900.1     VDFLGQKIDFTPFNGIISSLQDTASNVAKTISSQPPIKFSISNTRVESWLLTTYLDEDLR
                   ************************************************************

Csa6M512870.1      ISRGDGGSVFVLLKEGSSFLSL
Cucsa.044900.1     ISRGDGGSVFVLLKEGSSFLSL
                   **********************
```

Figure S1. Comparison of deduced amino acid sequences of CsaFBN1 (Csa6M512870.1) in var. Chinese long with that (Cucsa.044900.1) in var. Gy14. A different amino acid residue is indicated by red letter. The deduced amino acid sequences were aligned using ClustalW (http://www.genome.jp/tools/clustalw/) with default parameters.

```
Csa6M108600.1      MAKSLSLPSLPFIFPSSITSHLSHLPKISCSSISFQSQSAKRQLLTLISDQQRGLKTQKN
Cucsa.120630.1     MAKSLSLPSLPFISPSSITTHLSHLPKISCSSISIQSQSAKRQLLTLISDQQRGLKTQKN
                   ************* *****:**************:*************************

Csa6M108600.1      PQKLASIVKSIDHLASLGRNSVTTDDSLSATWRLLWTTEKEQLFIIEKAHLFGTRAGDVL
Cucsa.120630.1     PQKLASIVKSIDHLASLGRNSVTTDDSLSATWRLLWTTEKEQLFIIEKAHLFGTRAGDVL
                   ************************************************************

Csa6M108600.1      QVIDVEKKSLNNVITFPPDGVFFVRSNIEVASSQRVNFRFTSAVLRGKNWEIPLPPFGQG
Cucsa.120630.1     QVIDVEKKSLNNVITFPPDGVFFVRSNIEVASSQRVNFRFTSAVLRGKNWEIPLPPFGQG
                   ************************************************************

Csa6M108600.1      WFDTVYLDDEIRVVKDIRGDYLIVERAPYSWTE
Cucsa.120630.1     WFDTVYLDDEIRVVKDIRGDYLIVERAPYSWTE
                   *********************************
```

Figure S2. Comparison of deduced amino acid sequences of CsaFBN9 (Csa6M108600.1) in var. Chinese long with that (Cucsa.120630.1) in var. Gy14. Three different amino acid residues are indicated by red letters. The deduced amino acid sequences were aligned using ClustalW (http://www.genome.jp/tools/clustalw/) with default parameters.

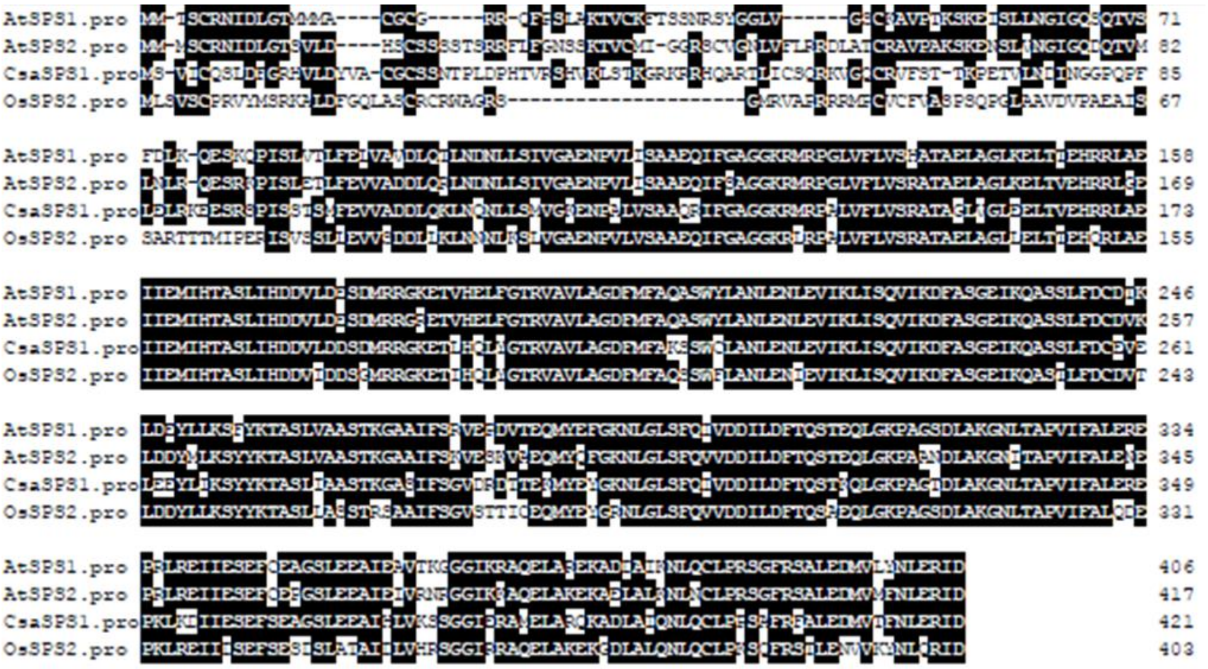

Figure S3. Protein sequence alignment among Arabidopsis SPS1, SPS2, cucumber SPS1, and rice SPS2. The deduced amino acid sequences were aligned using ClustalW (http://www.genome.jp/tools/clustalw/) with default parameters.

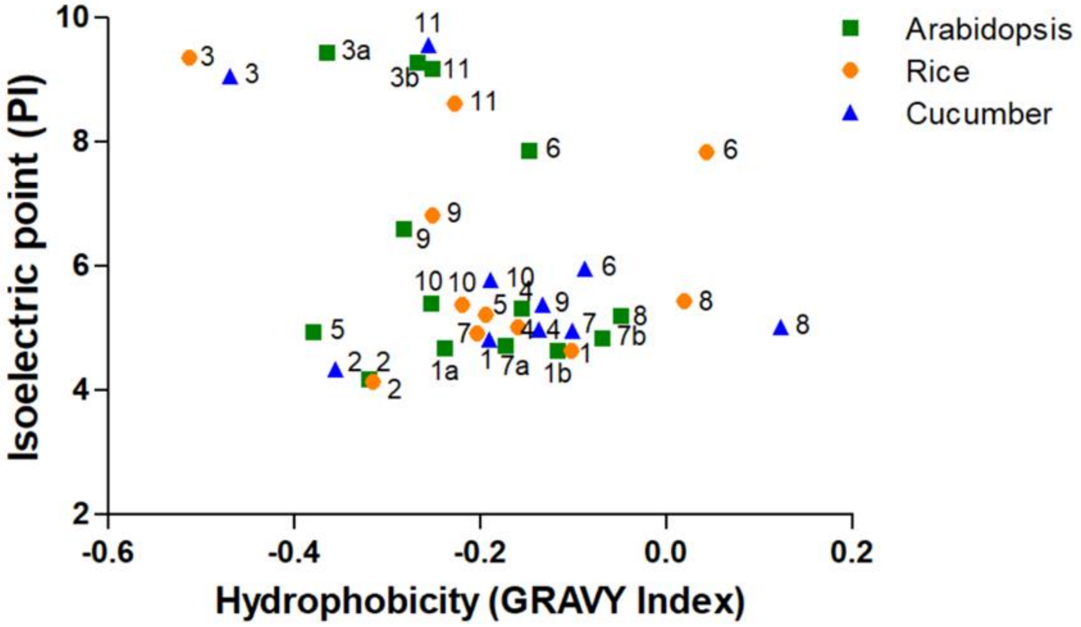

Figure S4. Physicochemical properties of FBN proteins from Arabidopsis, rice and cucumber. PI and hydrophobicity (GRAVY index) were measured for each FBN after removal of the chloroplast transit peptide using the ProtParam tool (ExpPASy).

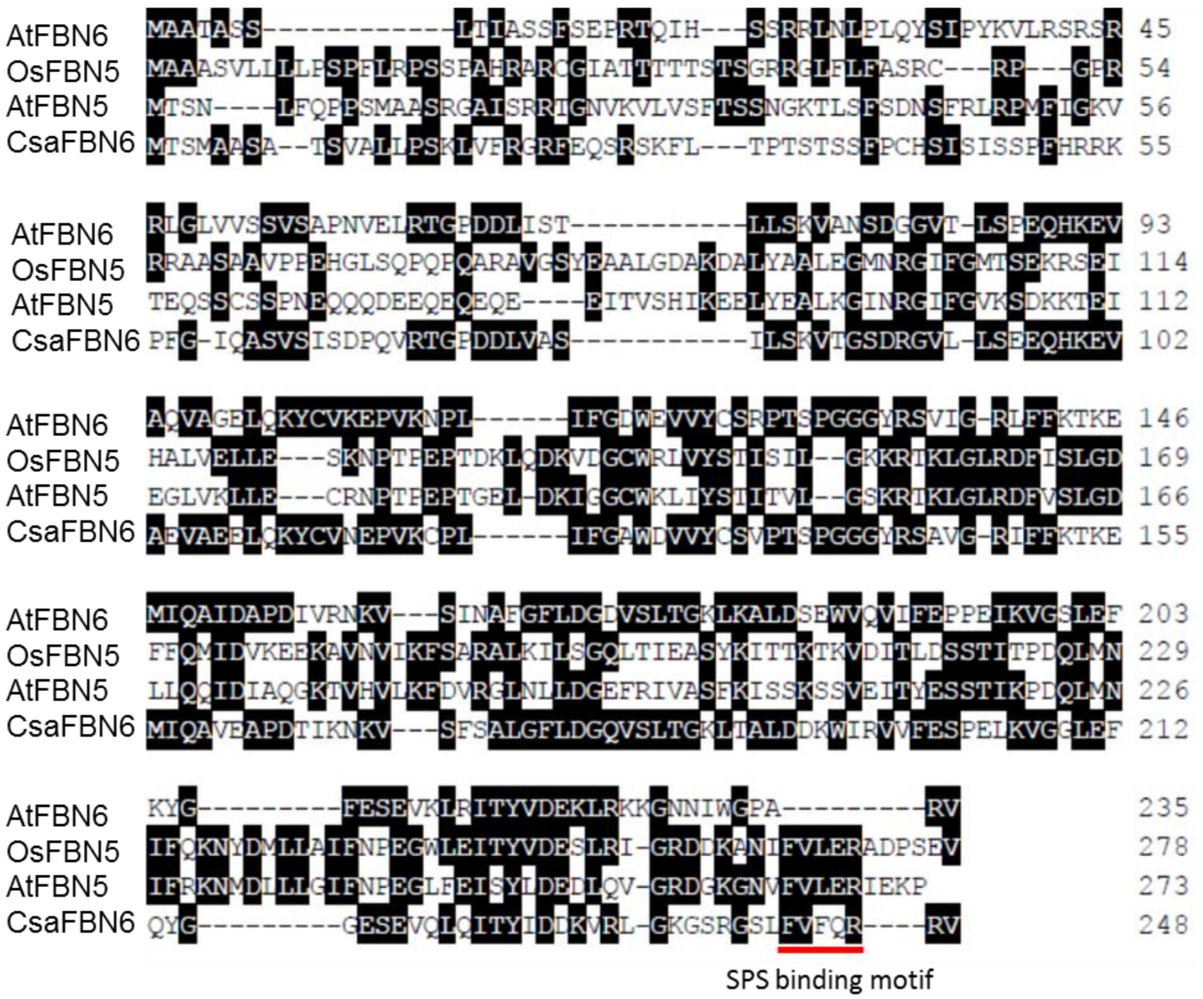

Figure S5. Protein sequence alignment among Arabidopsis FBN6, rice FBN5, Arabidopsis FBN5, and cucumber FBN6. The deduced amino acid sequences were aligned using ClustalW (http://www.genome.jp/tools/clustalw/) with default parameters. Possible SPS binding motif was underlined with red color.
